# Associations of adherence to Mediterranean-like diet pattern with incident rosacea: A prospective cohort study of government employees in China

**DOI:** 10.3389/fnut.2023.1092781

**Published:** 2023-02-02

**Authors:** Peng Chen, Ziye Yang, Zhihua Fan, Ben Wang, Yan Tang, Yi Xiao, Xiang Chen, Dan Luo, Shuiyuan Xiao, Ji Li, Hongfu Xie, Minxue Shen

**Affiliations:** ^1^Department of Dermatology, Xiangya Hospital, Central South University, Changsha, China; ^2^Hunan Engineering Research Center of Skin Health and Disease, Xiangya Hospital, Central South University, Changsha, China; ^3^Hunan Key Laboratory of Aging Biology, Xiangya Hospital, Central South University, Changsha, China; ^4^National Clinical Research Center for Geriatric Disorders, Xiangya Hospital, Central South University, Changsha, China; ^5^Furong Laboratory, Changsha, China; ^6^Department of Social Medicine and Health Management, Xiangya School of Public Health, Central South University, Changsha, China

**Keywords:** rosacea, Mediterranean diet, incidence rate, diet pattern analysis, anti-inflammatory

## Abstract

**Background:**

Despite of growing evidence on gastrointestinal comorbidities of rosacea, there was a lack of literatures regarding the role of diet on rosacea.

**Objectives:**

To investigate the relationship between adherence to a Mediterranean-like diet pattern and the risk of incident rosacea.

**Methods:**

This was a prospective cohort study of government employees aged >20 years conducted between January 2018 and December 2021 from five cities of Hunan province of China. At baseline, participants completed a food frequency questionnaire and participated in a skin examination. Presence of rosacea was determined by certified dermatologists. Subsequent skin examinations during follow-up were performed every one-year interval since the entry of the study. The Mediterranean diet score (MDS) was generated based on seven food groups (whole grains, red meats, fish, raw vegetables, legumes, fruits and nuts). Binary logistic regression models adjusted for potential confounders were used to estimate risks for incident rosacea.

**Results:**

Of the 3,773 participants who completed at least two consecutive skin examinations, 3,496 were eligible for primary analyses. With a total follow-up of 8,668 person-years, we identified 83 incident rosacea cases. After full adjustments for covariates, the MDS was associated a decreased risk of incident rosacea (aOR: 0.84, 95% CI: 0.72, 0.99; *P*_*trend*_ = 0.037 for 1-point increment of MDS). In subgroup analyses by body mass index (BMI), this inverse association was consistently observed in the lowest and medium tertiles of BMI (<24.5 kg/m^2^), but not in the highest tertile of BMI (≥24.5 kg/m^2^), with a significant interaction effect (*P* < 0.001).

**Conclusions:**

Our results suggested that adherence to a Mediterranean-like diet pattern might reduce the risk of incident rosacea among non-overweight individuals.

## Introduction

Rosacea is recognized as a common chronic inflammatory skin syndrome encompassing a variety of signs or symptoms, which affects 5.46% of the general population over the world (1, 2). Although the exact pathogenesis of rosacea still remains unclear, it's found that both genetic and environmental factors contributed to development of the disease (3). In one survey conducted by the National Rosacea Society, several dietary factors including heat-related, alcohol-related, capsaicin-related, and cinnamaldehyde-related, were reported as rosacea triggers by patients (4), which suggested the diet might play an important role in rosacea. What's more, epidemiological studies have demonstrated that compared to healthy subjects, patients with rosacea had a higher prevalence of gastrointestinal (GI) disease, such as *Helicobacter pylori* infection (HPI), small intestinal bacterial overgrowth (SIBO), and irritable bowel syndrome (IBS) (5, 6). These findings further emphasized the importance of carrying out dietary interventions to decrease the risk of GI comorbidities. However, there was a lack of literatures regarding the role of dietary factors on the development of rosacea, (4) with few studies only focusing on effects of single nutrients or food groups (7, 8).

The Mediterranean diet (MD) is a healthy diet pattern characterized as high intake of fruits, vegetables, whole grains, fish, legumes, and nuts; moderate consumption of alcohol; low intake of red or processed meats, and using olive oil as the main source of fat (9, 10). A meta-analysis has shown that greater adherence to MD was associated with decreased incidence of many chronic diseases (e.g., cancer, Parkinson's disease, and Alzheimer's disease) (11, 12). Epidemiology studies also demonstrated that adherence to the MD pattern reduced the mortality caused by a series of metabolic diseases, such as cardiovascular disease and type 2 diabetes (13–15). The underlying mechanisms could partially be explained by the anti-inflammatory capacity of MD (16). Meanwhile, the MD pattern had also shown protective roles on severity or development of certain inflammatory skin disorders like acne and psoriasis (17–20). Similarly, higher levels of pro-inflammatory serum markers like C-reaction protein (CRP) in rosacea patients indicated the involvement of both cutaneous and systemic inflammation in the pathogenesis of rosacea (21–23).

Combining this evidence, we hypothesized that MD might also have an effect on the onset of rosacea. The objective of the current study was therefore to investigate the relationship between a score reflecting adherence to MD and the risk of incident rosacea in a prospective cohort of government employees. The traditional MD score was mainly applied in Mediterranean countries like Greece or for samples of elderly participants (24). To adapt the score to Chinese population, we selected the alternate Mediterranean Diet (aMed) score established by Fung et al. to evaluate adherence to MD in our study (25).

## Materials and methods

### Study design and population

This was a population-based prospective cohort study conducted from January 2018 to December 2021 in five cities (Changsha, Zhuzhou, Xiangtan, Huaihua, and Changde) of Hunan province, China. On the basis of informed consent, a total of 11,523 government employees aged >20 years from 25 organizations were enrolled. At baseline, they completed a questionnaire collecting information on demographic, socioeconomic, and lifestyle behaviors and participated in a dermatological physical examination at the same time. Subsequent skin examinations were performed every one-year interval since their enrollment of the study to update their skin health status during the follow-up period.

The follow up began on the date the questionnaires were returned or the first physical examinations were performed (baseline). Participants contributed person-time from baseline until the date of the first-time rosacea diagnosis during follow-up or the date of last-time physical examination (due to loss to follow up or the end of the study), whichever came first.

In the current study, we only included participants who completed at least two consecutive dermatological physical examinations (*n* = 3773). Thus, the follow-up time of participants ranged from 1 to 4 years. The study procedures were approved by the institutional research ethics boards of Xiangya School of Public Health, Central South University (XYGW-2016-10).

### Outcome ascertainment

The presence of rosacea was determined by certified dermatologists from local tertiary hospitals in each dermatological physical examination. In the field survey, clinical manifestation, disease history, and family history of participants were inquired, and dermatologists made an accurate diagnosis of rosacea based on the diagnostic criteria established by the National Rosacea Society Expert Committee in 2017 (at least 1 diagnostic or 2 major phenotypes were required) (2).

### Dietary assessment and Mediterranean diet score calculation

The weekly average frequency of dietary intake for different food groups over the preceding year of enrollments was assessed by a self-reported semi-quantitative food frequency questionnaire (FFQ) at baseline. Each food groups had 5 possible frequency of intake responses in the FFQ: rarely, <1 time/week, 1–3 days/week, 4–6 days/week and every day. The Mediterranean diet score (MDS) applied in this study was adopted from the aMed score posted by Fung et al. (25). As alcohol drinking was associated with increased risks of incident rosacea even under low doses of daily intake, it was not included in the MDS calculation (26). Due to the availability of data, ratio of monounsaturated to saturated fat was also excluded. Eventually, our MDS consisted of seven components of food groups: whole grains, red meats, fish, raw vegetables, legumes, fruits, and nuts. Each component was assigned with point 0 or 1 using sex-specific medians of food intake frequency categories as cut-offs. For presumed beneficial food groups (whole grains, fish, raw vegetables, legumes, fruits, and nuts), participants whose intake was below the median categories received a point of 0, and a point of 1 otherwise. For presumed detrimental food groups (red meats), participants whose intake was below the median categories received a point of 1, and a point of 0 otherwise. Thus, the possible MDS ranged from 0 to 7, with higher score representing closer adherence to the Mediterranean diet pattern. We additionally separated participants by approximately tertiles of MDS into three groups: low (0–3), medium (4–5), and high (6–7).

### Covariates

Information on socioeconomic characteristics (education level, annual household income) and behaviors (smoking status, alcohol drinking status, frequency of sunbath, frequency of physical exercise) was collected by questionnaires at baseline. New variables were created when including cigarette smoking and alcohol drinking for adjustments in multivariable models by further including information on daily average consumption of cigarettes and frequency of drinking per week in current smokers and drinkers. The body mass index (BMI) was calculated by dividing individuals' weight in kilograms by the square of their height in meters which were obtained from physical examination.

### Statistical analysis

Descriptive statistics were performed to summarize participants' characteristics at baseline stratified by categories of MDS. Continuous variables were presented as mean ± standard deviation (SD) and categorical variables were presented as number (%). Between-group differences were tested by ANOVA or chi-square test for continuous and categorical variables. We used binary logistic regression models to assess the association between incident rosacea and variables of interests and calculated the odds ratio (OR) and 95% confidence interval (CI).

First, we evaluated the association of incident rosacea with each component of food groups in the MDS separately. Those food intake frequency groups with small sample sizes were merged into one group where appropriate to achieve approximately tertiles and the lowest tertiles were treated as reference groups. Then, we investigated the association of MDS with risks of incident rosacea using MDS categories with lowest (0–3) adherence to MD as reference. A *P*-value for trend across MDS categories was calculated by treating MDS as a continuous variable. We also applied natural cubic splines to examine potential non-linear or linear relationships between MDS and incidence of rosacea. As the obesity was reported as a risk factor for incident rosacea in previous study, (27) we additionally tested effect modification by BMI by adding an interaction term between MDS and BMI into logistic models. We then stratified the analyses by tertiles of BMI. Subgroup analyses by other potential covariates were also performed.

To test the robustness of the primary analyses, we performed sensitivity analyses by (1) excluding participants who made significant changes on dietary or lifestyle habits last year before the entry of the study, or (2) excluding participants with prevalent or incident acne vulgaris, contact dermatitis or seborrheic dermatitis which were considered as differential diagnoses of rosacea for patients with skin of color (28).

We used principal component analysis (PCA) to extract components of intake frequency of other dietary factors not associated with calculation of MDS in our FFQ (rice, pasta, poultry, eggs, dairy products, pickles, deserts, and marinated or smoked fish) with eigenvalues >1, for the purpose of addressing the collinearity issues among dietary factors when being included in multivariate models. All basic models were adjusted by age and sex, and the fully adjusted models were further adjusted by education level, annual household income, BMI, cigarette smoking, alcohol drinking, sunbath, physical exercise, and specially, the principal components of other dietary factors not associated with calculation of MDS to reduce residual confounding. Participants with missing data on dietary intake frequency associated with calculation of MDS was eliminated, while missing data on covariates was imputed by multiple imputation.

All statistical analyses were performed using R software (version 4.1.3). A *P* < 0.05 was considered significant.

## Results

### Baseline characteristics stratified by MDS categories

After excluding participants with unavailable follow-up time (*n* = 68), prevalent rosacea (*n* = 51), and missing data on dietary intake frequency (*n* = 158), there were a total of 3,496 participants included in the primary analyses (Supplementary Figure S1). During a median follow-up of 2 years (interquartile range: 2–3 years; 8,668 total person-years), we identified 83 incident rosacea cases (incidence rate: 9.58 per 1,000 person-years). The baseline characteristics of participants stratified by MDS categories were shown in Table 1. Among 3,496 eligible participants, 23.3, 45.8, and 30.9% of them were categorized into low, medium, and high adherence to Mediterranean diet pattern groups. Compared to participants with either low or medium adherence to MD, those in the high adherence group had older age, higher education level, annual household income, and frequency of physical exercise, and lower rate of current smokers (all *p*-values <0.05). We extracted three components from other dietary factors not associated with calculation of MDS, accounting for 33.6, 16, and 13% of the total variance, respectively (Supplementary Figure S2).

**Table 1:** Baseline characteristics of the study population according to MDS categories (*n* = 3496).

**Characteristics**	**Mediterranean diet score**			***P-*value**	**Missing value**
	**Low (0-3)**	**Medium (4-5)**	**High (6-7)**		
Participants, *n* (%)	816 (23.3)	1602 (45.8)	1078 (30.9)		
Age (year), mean ± SD	38.05 ± 9.08	38.50 ± 9.25	39.53 ± 9.30	0.001	
Sex, *n* (%)				0.072	
Male	354 (43.4)	676 (42.2)	416 (38.6)		
Female	462 (56.6)	926 (57.8)	662 (61.4)		
BMI (Kg/m^2^), mean ± SD	23.39 ± 3.75	23.46 ± 3.58	23.47 ± 3.71	0.866	1
Annual household income (CNY), *n* (%)				<0.001	1
<50,000	121 (14.8)	177 (11.0)	119 (11.0)		
50,000 ~ 100,000	241 (29.6)	398 (24.8)	269 (25.0)		
100,000 ~ 200,000	286 (35.1)	617 (38.5)	392 (36.4)		
>200,000	167 (20.5)	410 (25.6)	298 (27.6)		
Education level, *n* (%)				<0.001	384
High school and below	55 (7.7)	77 (5.4)	45 (4.6)		
Undergraduate degree	461 (64.3)	772 (54.3)	507 (52.2)		
Postgraduate degree and above	201 (28.0)	574 (40.3)	420 (43.2)		
Smoking status, *n* (%)				0.003	193
Non-smoker	640 (83.4)	1307 (86.4)	918 (89.6)		
Current smoker	109 (14.2)	175 (11.6)	85 (8.3)		
Past smoker	18 (2.3)	30 (2.0)	21 (2.1)		
Alcohol drinking status, *n* (%)				0.242	
Non-drinker	700 (85.8)	1413 (88.2)	961 (89.1)		
Current drinker	108 (13.2)	173 (10.8)	107 (9.9)		
Past drinker	8 (1.0)	16 (1.0)	10 (0.9)		
Frequency of physical exercise, *n* (%)				<0.001	263
Rarely	456 (61.3)	719 (48.1)	359 (36.1)		
1–2 times/week	176 (23.7)	396 (26.5)	316 (31.8)		
≥ 3 times/week	112 (15.1)	380 (25.4)	319 (32.1)		

CNY, Chinese Yuan; MDS, Mediterranean diet score; SD, standard deviation.

### Food groups and incident rosacea

Associations between intakes of each food groups from MDS and incident rosacea were reported in Table 2. Taken individually, we observed that the intake of whole grains with ≥4 days/week was associated with a decreased risk of incident rosacea compared to the lowest intake frequency group (<1 time/week) in both basic model and fully adjusted model (aOR: 0.37; 95%CI: 0.17, 0.84). However, no significant association was found for other food groups in fully adjusted models.

**Table 2:** Associations of each food groups from MDS with incident rosacea (*n* = 3496).

	**Total no. of participants**	**No. of cases of rosacea/person-years**	**Incidence rate (per 1000 person-years)**	**Model 1**		**Model 2**	
				**aOR (95%CI)**	***P***	**aOR (95%CI)**	***P***
Total	3,496	83/8668	9.58				
**Whole grains**							
<1 time/week	1,489	44/3711	11.86	Reference		Reference	
1–3 days/week	1,308	31/3243	9.56	0.73 (0.46, 1.17)	0.190	0.73 (0.45, 1.18)	0.196
≥4 days/week	699	8/1714	4.67	0.37 (0.17, 0.79)	0.011	0.37 (0.17, 0.84)	0.017
**Red meats**							
≤3 days/week	1,164	35/2907	12.04	Reference		Reference	
4-6 days/week	878	15/2199	6.82	0.60 (0.32, 1.11)	0.102	0.67 (0.35, 1.26)	0.209
Everyday	1,454	33/3562	9.26	0.83 (0.51, 1.34)	0.438	0.96 (0.55, 1.67)	0.892
**Fish**							
Rarely	506	18/1222	14.73	Reference		Reference	
<1 time/week	1,378	33/3483	9.47	0.62 (0.34, 1.12)	0.111	0.69 (0.37, 1.27)	0.235
≥1 day/week	1,612	32/3963	8.07	0.54 (0.30, 0.97)	0.041	0.61 (0.32, 1.17)	0.137
**Raw vegetables**							
≤3 days/week	457	15/1182	12.69	Reference		Reference	
4-6 days/week	681	19/1719	11.05	0.89 (0.44, 1.77)	0.732	0.90 (0.44, 1.84)	0.781
Everyday	2,358	49/5767	8.50	0.64 (0.35, 1.19)	0.158	0.68 (0.35, 1.33)	0.259
**Legumes**							
<1 time/week	877	26/2147	12.11	Reference		Reference	
1–3 days/week	1,659	40/4150	9.64	0.77 (0.46, 1.27)	0.305	0.77 (0.46, 1.31)	0.343
≥4 days/week	960	17/2371	7.17	0.58 (0.31, 1.08)	0.084	0.59 (0.29, 1.21)	0.149
**Fruits**							
≤3 days/week	1,512	36/3720	9.68	Reference		Reference	
4-6 days/week	779	15/1947	7.70	0.66 (0.36, 1.22)	0.182	0.73 (0.39, 1.37)	0.322
Everyday	1,205	32/3001	10.66	0.89 (0.54, 1.45)	0.628	1.05 (0.61, 1.80)	0.854
**Nuts**							
<1 time/week	1,324	36/3249	11.08	Reference		Reference	
1-3 days/week	1,459	29/3643	7.96	0.66 (0.40, 1.08)	0.096	0.72 (0.43, 1.22)	0.222
≥4 days/week	713	18/1776	10.14	0.85 (0.47, 1.52)	0.575	1.00 (0.51, 1.98)	0.990

Model 1: Adjusted by age and sex. Model 2: Further adjusted by education level, annual household income, BMI (continuous), cigarette smoking (never, past, or current smoking of 1–14, 15–20, or >20 cigarettes/d), alcohol drinking (rarely, past, or current drinking of 1, 2-4, or ≥ 5 times/week), sunbath (rarely, sometimes, and frequently), frequency of physical exercise, and principal components of other dietary factors.

### Mediterranean diet score and risks of incident rosacea

**Figure 1:**
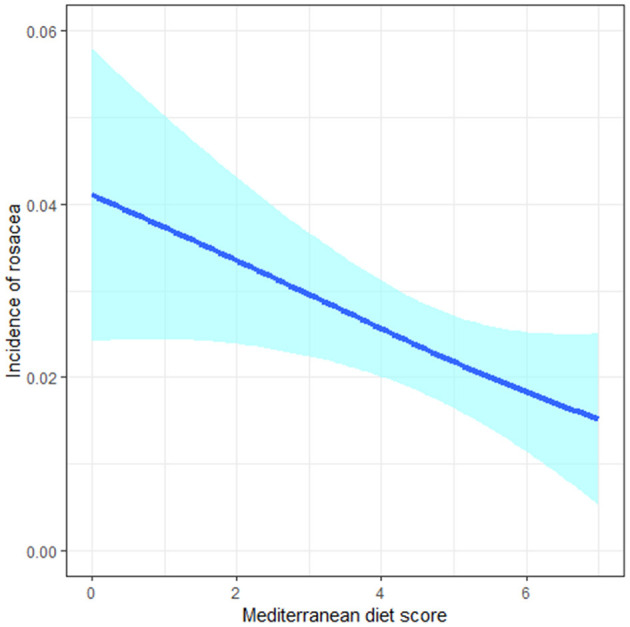
The negative linear relationship between the Mediterranean diet score and incidence of rosacea in cubic splines.

In the whole study population, we observed an inverse association between MDS and the risk of incident rosacea in both basic and fully-adjusted models (aOR: 0.84, 95%CI: 0.72, 0.99; *P*_*trend*_ = 0.037 for 1-point increment of MDS) (Table 3). Cubic splines revealed a negative linear relationship between MDS and the incidence of rosacea (Figure 1).

**Table 3:** Associations of Mediterranean diet score with incident rosacea (*n* = 3496).

	**Mediterranean diet score**			**Per 1-point increment of MDS**	***P-value* for trend**
	**Low (0-3)**	**Medium (4-5)**	**High (6-7)**		
No. of Cases of Rosacea/Person-Years	29/2029	33/3974	21/2665		
Incidence Rate (Per 1000 Person-years)	14.29	8.30	7.88		
Age- and sex-adjusted OR (95% CI)	Reference	0.56 (0.34, 0.94)	0.52 (0.29, 0.92)	0.83 (0.72, 0.96)	0.010
Multivariate adjusted OR (95% CI) ^*^	Reference	0.58 (0.34, 1.00)	0.55 (0.28, 1.06)	0.84 (0.72, 0.99)	0.037

^*^Models were further adjusted by education level, annual household income, BMI (continuous), cigarette smoking (never, past, or current smoking of 1–14, 15–20, or >20 cigarettes/d), alcohol drinking (rarely, past, or current drinking of 1, 2-4, or ≥ 5 times/week), sunbath (rarely, sometimes, and frequently), frequency of physical exercise, and principal components of other dietary factors.

### Subgroup analysis

**Figure 2:**
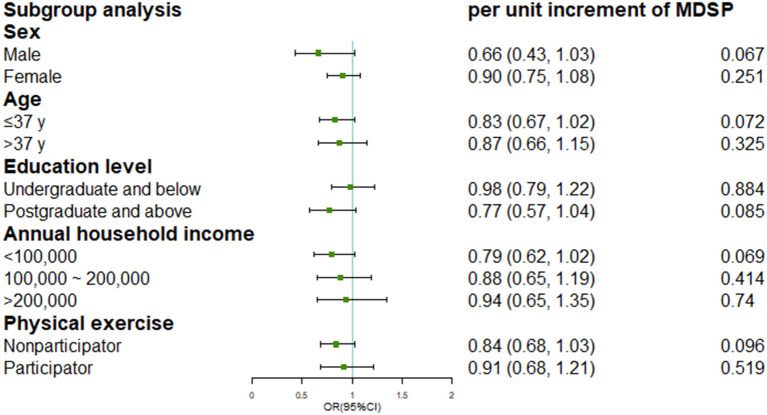
Subgroup analysis for the association of Mediterranean diet score and incident rosacea by potential covariates.

Since an interaction effect between BMI and MDS was detected (*P*_*interaction*_ <0.001), we further stratified the analyses by tertiles of BMI shown in Table 4 (two participants eliminated with missing value or extremely low value on BMI). Among participants in tertile 1 (BMI <21.8) and tertile 2 (BMI range: 21.8–24.5), we consistently found inverse associations between MDS and incident rosacea in fully-adjusted models (*P*_*trend*_ = 0.018 and 0.023, respectively). However, no significant protective effects of MDS were observed in those with highest tertile of BMI (tertile 3: BMI ≥ 24.5) in both the basic model and fully-adjusted model. Subgroup analysis by other covariates was presented in Figure 2.

**Table 4:** Subgroup analysis for the associations of MDS with incident rosacea by tertiles of BMI (*n* = 3494).

	**Total no. of participants**	**No. of cases of rosacea/person-years**	**Incidence rate (per 1000 person-years)**	**Model 1**		**Model 2**	
				**aOR (95%CI)**	***P***	**aOR (95%CI)**	***P***
**Tertile 1 (**<**21.8)**	1,164	36/2993	12.03				
Low (0–3)	278	14/730	19.18	Reference		Reference	
Medium (4–5)	539	17/1388	12.25	0.60 (0.29, 1.23)	0.163	0.66 (0.30, 1.48)	0.318
High (6–7)	347	5/875	5.71	0.26 (0.09, 0.74)	0.012	0.31 (0.10, 0.99)	0.047
Per 1-point increment of MDS				0.72 (0.58, 0.89)		0.74 (0.58, 0.95)
*P_*trend*_*				0.002		0.018
**Tertile 2 (21.8-24.5)**	1,165	26/2843	9.15				
Low (0–3)	268	11/651	16.90	Reference		Reference	
Medium (4–5)	512	9/1255	7.17	0.42 (0.17, 1.02)	0.056	0.36 (0.14, 0.95)	0.038
High (6–7)	385	6/937	6.40	0.36 (0.13, 0.99)	0.047	0.27 (0.08, 0.91)	0.035
Per 1-point increment of MDS				0.76 (0.59, 0.96)		0.71 (0.53, 0.95)	
*P_*trend*_*				0.022		0.023	
**Tertile 3 (≥24.5)**	1,165	21/2826	7.43				
Low (0–3)	270	4/648	6.17	Reference		Reference	
Medium (4–5)	549	7/1325	5.28	0.79 (0.23, 2.76)	0.717	0.93 (0.25, 3.45)	0.912
High (6–7)	346	10/853	11.72	1.77 (0.54, 5.76)	0.345	2.35 (0.62, 8.97)	0.210
Per 1-point increment of MDS				1.26 (0.90, 1.75)		1.36 (0.95, 1.93)	
*P_*trend*_*				0.175		0.090	

Model 1: Adjusted by age and sex. Model 2: Further adjusted by education level, annual household income, cigarette smoking (never, past, or current smoking of 1–14, 15–20, or >20 cigarettes/d), alcohol drinking (rarely, past, or current drinking of 1, 2-4, or ≥ 5 times/week), sunbath (rarely, sometimes, and frequently), frequency of physical exercise, and principal components of other dietary factors.

### Sensitivity analysis

The results of primary analyses remained stable when excluding participants with significant lifestyle or dietary modifications over the preceding year, or excluding participants with skin disorders that were differential diagnoses of rosacea (Supplementary Table S1).

## Discussion

In this population-based prospective cohort of Chinese government employees, we observed an inverse association between adherence to MD and risks of incident rosacea. This association was consistently present in groups with lowest and medium tertiles of BMI (BMI <24.5 Kg/m^2^), but not in group with highest tertile of BMI (BMI ≥ 24.5 Kg/m^2^). To our knowledge, this was the first study to report a positive association between MD and rosacea.

In this study, the incidence rate of rosacea was 9.58 per 1,000 person-years. Previously, only one study from U.K.-based General Practice Research Database had reported an incidence rate of rosacea (1.65 per 1,000 person-years), which was notably lower than our study (1, 29). However, the ascertainment of rosacea from this database was performed by general practitioners in primary care and diagnoses were primarily based on visible symptoms due to lacking diagnostic guidelines, thus leading to underestimate of the incidence rate for missing cases with mild rosacea (29). By contrast, presence of rosacea in this study was determined by certified dermatologists in field survey for all enrolled participants based on the latest diagnostic criteria combining presenting cutaneous signs and personal history, which allowed us to assume a higher diagnostic accuracy (2). Moreover, to address the concern of misdiagnosis, we also performed sensitivity analysis by excluding participants with skin disorders that were considered as differential diagnoses of rosacea.

As mentioned before, prior studies investigating the role of diet on rosacea mainly focused on single nutrients or food components. For instance, a case-control study with 1,347 rosacea patients and 1,290 controls reported a positive correlation between high-frequency intake of fatty food and tea and risks of rosacea, while high-frequency intake of dairy products showed a negative correlation (7). In our analyses of single food components, we only observed a significant inverse association between intakes of whole grains and incident rosacea. However, there seemed to be a negative but non-significant correlation between higher intake frequency of other beneficial components of MD and the incidence of rosacea. This could partially be explained that the effect of single food components might be too small to detect among individuals, while a dietary pattern approach examining cumulative effects of multiple nutrients or food components was feasible to identify overall effects. What's more, dietary pattern analysis considered the issues of complex interactions and correlations among nutrients (30).

Although the mechanism of health benefits of MD had not been fully understood, several hypotheses could be considered when interpreting our results. First, the MD exerted antioxidative properties. It's known that oxidative stress plays a role in the pathogenesis of rosacea. Individuals with rosacea were confirmed to have higher serum peroxide levels and lower serum antioxidative potential levels (31). Takci et al. (32) also demonstrated that rosacea patients had decreased activity of Paraoxonase-1 (PON1), an antioxidant enzyme, and higher levels of serum lipid hydroperoxide (32). Multiple components of MD, including nuts, fruits, and vegetables, have been proven effective in increasing PON1 activity and reducing lipid peroxidation (33). Second, the MD pattern exhibited an anti-inflammatory capacity. The elevated levels of chemokines and CRP in serum (21, 34). and pro-inflammatory cytokines (IL-8, IL-1β, TNF-α) in skin lesions implied that rosacea was not only a cutaneous inflammatory skin disorder but also with low-grade systemic inflammation (22, 35). Interestingly, diet intervention studies revealed inverse associations between MD and almost all inflammatory biomarkers which indicated the capacity of MD to reduce low-grade inflammation (36). This was potentially mediated by modulation of the gastrointestinal (GI) microbiota. Altered GI microbiota, a pivotal element of gut-skin axis, has been observed by emerging studies in rosacea and might be responsible for increased risks of GI comorbidities in rosacea (37, 38). Multiple nutrients from MD, including polyphenols, polyunsaturated fatty acids (PUFAs) and fiber, contribute to modulations of the gut microbiota both in the aspects of diversity and inflammatory response (39). For example, the ω-3 PUFAs, rich in fish and nuts, could induce increases of several short-chain fatty acid-producing intestinal bacteria thus leading to suppression of inflammation, (40, 41) and was reported effective in the treatment of ocular rosacea (42). In addition, the MD was also associated with an increased abundance of fiber-degrading bacteria in GI microbiota and lower levels of intestinal inflammation (43). We hypothesized that a chronic subclinical inflammation caused by obesity might counterbalance the anti-inflammatory capacity of MD, which could explain the interaction effects between BMI and MDS (16, 27).

Our study encompassed several strengths and limitations. The strengths included the prospective study design, the dermatologists-based rosacea diagnoses, annually-renewable skin health status, and the availability of various epidemiological factors which allowed us to adjust for potential confounders and performed sensitivity analyses based on multiple conditions. In the meanwhile, we also acknowledged the existence of several limitations in our study. First, this study had a high rate of loss to follow-up (referring to those excluded for only attending skin examinations at baseline) and this population represented a lower level of education and income compared to those included in primary analyses (Supplementary Table S2). As a result, even though we adjusted the models with education, income and lifestyle factors, the selection bias was inevitable, and it should be interpreted with caution when generalizing our results to the public population. Second, our study was limited by short periods of follow-up and few cases of incident rosacea. However, a short-time study duration ensured the stability of dietary habits of participants during follow-up. Moreover, we also applied sensitivity analyses by excluding participants with dietary modifications to minimize the measurement error of dietary intake by FFQ. Third, it's worth noting that due to the availability of data, our MDS didn't include the variable of monounsaturated fat (MUFA) to saturated fat (SFA) ratio thus only representing a Mediterranean-like diet pattern. Nevertheless, this parameter could partially be reflected by the intakes of nuts and meat products which were rich in MUFA and SFA, respectively (44, 45). The efficacy of unsaturated fat in rosacea treatment also allowed us to assume an underestimate of protective effects of the Mediterranean diet on rosacea (42).

## Conclusion

In summary, this study indicated that adherence to a Mediterranean-like diet pattern was associated with lower risks of incident rosacea among non-overweight individuals. Our results needed to be verified in other population-based prospective cohort studies with larger sample sizes, longer follow-up periods and quantitative measurement on foods or nutrients intake.

## Data availability statement

The raw data supporting the conclusions of this article will be made available by the authors, without undue reservation.

## Ethics statement

The studies involving human participants were reviewed and approved by the Ethics Committee of Xiangya Hospital, Central South University. The patients/participants provided their written informed consent to participate in this study.

## Author contributions

PC drafted the manuscript and analyzed the data. ZY, ZF, BW, YT, YX, DL, and SX participated in the field investigation. MS and HX designed the study. MS, HX, XC, and JL obtained the funding. All authors participated in the data collection, critically revised the manuscript, and gave final approval to the version submitted for publication.
