# Supplementary figures and images for: Associations of adherence to Mediterranean-like diet pattern with incident rosacea: A prospective cohort study of government employees in China
Source: Front Nutr. 2023 Feb 2;10:1092781. doi: 10.3389/fnut.2023.1092781 (PMC9932686; doi:10.3389/fnut.2023.1092781)

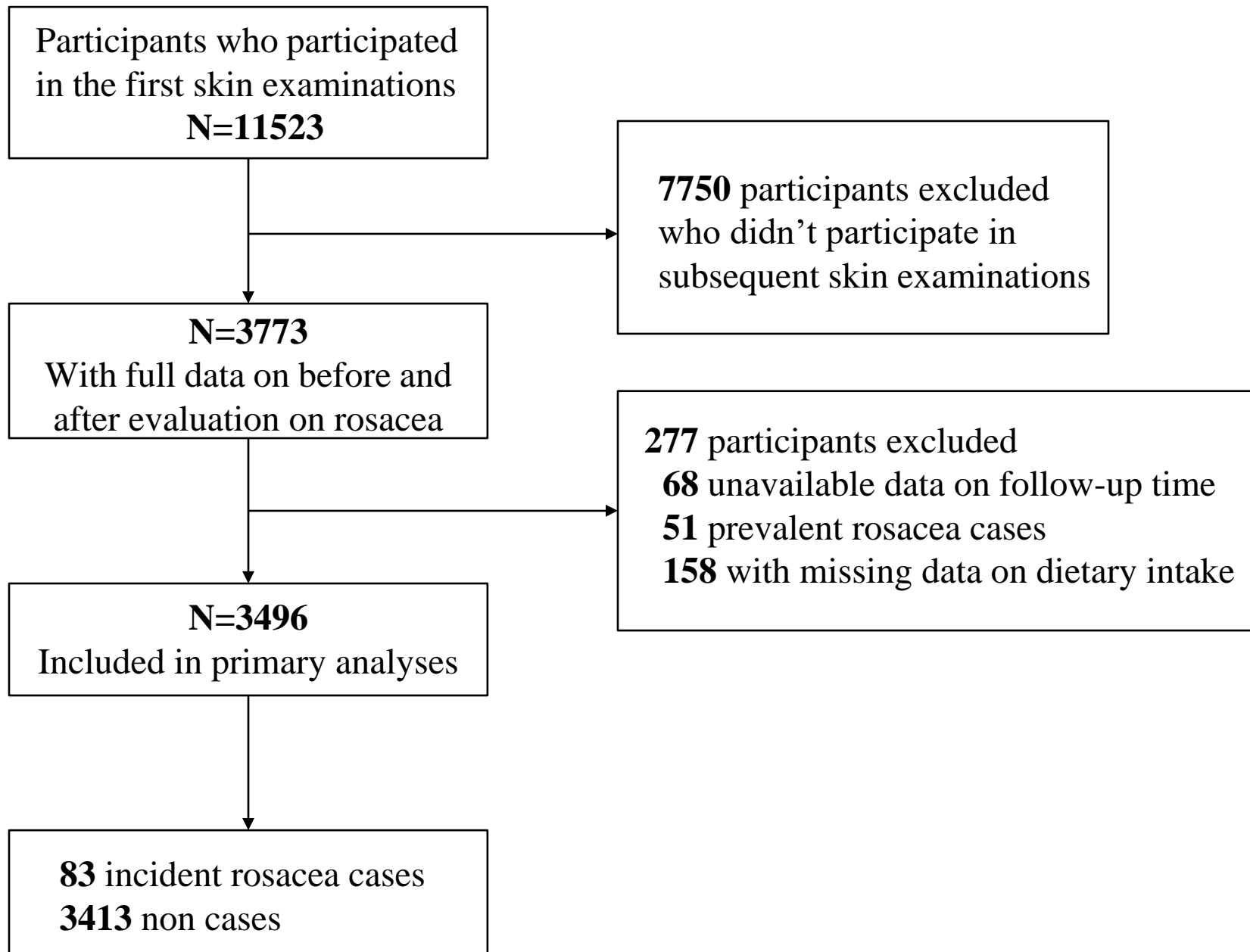

**Figure S1. Study flow chart**

Supplement: Supplementary file 1 [file Image_1.pdf]

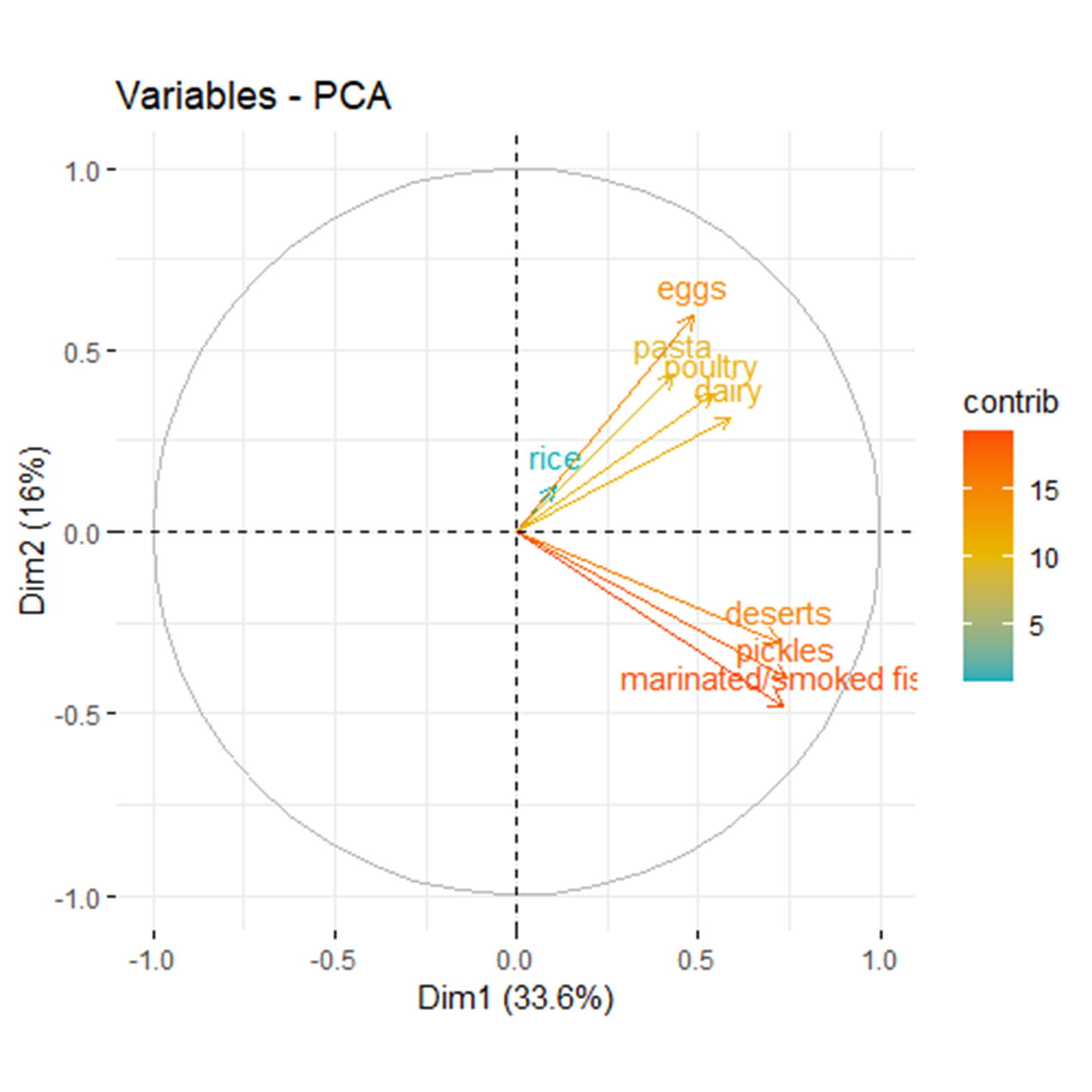

Supplement: Supplementary file 2 [file Image_2.tiff]
