# Supplementary material for: Associations of adherence to Mediterranean-like diet pattern with incident rosacea: A prospective cohort study of government employees in China
Source: Front Nutr. 2023 Feb 2;10:1092781. doi: 10.3389/fnut.2023.1092781 (PMC9932686; doi:10.3389/fnut.2023.1092781)
Supplement: Supplementary file 3 [file Data_Sheet_1.docx]

**Table S1. Sensitivity analysis for the association of Mediterranean diet score with incident rosacea**

|  | Mediterranean diet score | | | Per 1-point increment of MDS | *P-value* for trend |
| --- | --- | --- | --- | --- | --- |
|  | Low (0-3) | Medium (4-5) | High (6-7) |  |  |
| **Excluding participants who made significant changes on dietary or lifestyle habits last year (N=246)** | | | | |  |
| No. of Cases of Rosacea/Person-Years | 25/1829 | 27/3674 | 20/2527 |  |  |
| Incidence Rate (Per 1000 Person-years) | 13.67 | 7.35 | 7.91 |  |  |
| Age- and sex-adjusted OR (95% CI) | Reference | 0.52 (0.30, 0.90) | 0.54 (0.30, 0.98) | 0.83 (0.72, 0.97) | 0.016 |
| Multivariate adjusted OR (95% CI) ^*^ | Reference | 0.53 (0.30, 0.96) | 0.56 (0.28, 1.11) | 0.84 (0.70, 1.00) | 0.044 |
| **Excluding participants with prevalent or incident acne, contact dermatitis, or seborrheic dermatitis (N=270)** | | | | |  |
| No. of Cases of Rosacea/Person-Years | 25/1859 | 29/3656 | 18/2446 |  |  |
| Incidence Rate (Per 1000 Person-years) | 13.45 | 7.93 | 7.36 |  |  |
| Age- and sex-adjusted OR (95% CI) | Reference | 0.57 (0.33, 0.98) | 0.51 (0.27, 0.94) | 0.83 (0.71, 0.96) | 0.013 |
| Multivariate adjusted OR (95% CI) ^*^ | Reference | 0.58 (0.33, 1.04) | 0.52 (0.26, 1.07) | 0.83 (0.70, 0.99) | 0.038 |

^*^: Models were further adjusted by education level, annual household income, BMI (continuous), cigarette smoking (never, past, or current smoking of 1–14, 15–20, or >20 cigarettes/d), alcohol drinking (rarely, past, or current drinking of 1, 2-4, or ≥ 5 times/week), sunbath (rarely, sometimes, and frequently), frequency of physical exercise, and principal components of other dietary factors.

**Table S2. Baseline characteristics comparison between the study population and those lost to follow up**

| Characteristics | Study population (N=3773) | Lost to follow up (N=7750) | *P-value* |
| --- | --- | --- | --- |
| Female, n (%) | 2243 (59.4) | 4567 (58.9) | 0.608 |
| Age (year), mean ± SD | 38.65 ± 9.22 | 39.18 ± 11.73 | 0.018 |
| BMI (Kg/m^2^), mean ± SD | 23.46 ± 3.79 | 23.79 ± 5.14 | < 0.001 |
| Annual household income (CNY), n (%) |  |  | < 0.001 |
| <50,000 | 438 (12.1) | 1171 (17.3) |  |
| 50,000 ~ 100,000 | 923 (25.6) | 2080 (30.7) |  |
| 100,000 ~ 200,000 | 1339 (37.1) | 2280 (33.6) |  |
| >200,000 | 908 (25.2) | 1255 (18.5) |  |
| Education level, n (%) |  |  | < 0.001 |
| High school and below | 189 (5.9) | 512 (8.6) |  |
| Undergraduate degree | 1801 (56.1) | 3973 (66.7) |  |
| Postgraduate degree and above | 1225 (38.1) | 1472 (24.7) |  |
| Smoking status, n (%) |  |  | 0.126 |
| Non-smoker | 2967 (86.9) | 5960 (85.6) |  |
| Current smoker | 377 (11.0) | 863 (12.4) |  |
| Past smoker | 71 (2.1) | 136 (2.0) |  |
| Alcohol drinking status, n (%) |  |  | 0.547 |
| Non-drinker | 3182 (88.2) | 6351 (88.2) |  |
| Current drinker | 392 (10.9) | 767 (10.6) |  |
| Past drinker | 34 (0.9) | 84 (1.2) |  |

CNY: Chinese Yuan; SD: standard deviation.
